# Dr. J. A. Chapple

**Published:** 1906-06

**Authors:** B. H. Teague


### OBITUARY.

Dr. J. A. Chapple died suddenly, May 16th, 1906, of heart failure, superinduced by asthma.

John Abner Chapple, D. D. S.

He had taken a seat at a table for his lunch in the restaurant of his office building, when he fell, unconscious and in a few minutes expired. He was a sufferer for many years from the malady which terminated in his death. His remains were interred at La Grange, Ga., beside two of his children who had died in years past. Dr. Chapple was born October 23, 1854, at Greensboro, Ga. When a child his parents, Robert and Martha Francis Chapple, moved to Athens, Ga., where he grew up to manhood being educated in the schools of that city. He was a student of dentistry under Dr. J. A. Lawrence also of Athens. He graduated from the Baltimore College of Dental Surgery, class of 1874. Practiced at La Grange, Ga., until 1892, when he moved to Atlanta, Ga. Dr. Chapple was an active member of the Georgia State Dental Society and was one of its presidents, also was in active membership with the National Dental Association and its Southern Branch and the Chapin Harris Dental Society; and was an honorary member of numerous other dental bodies. For many years he was one of the faculty of the Atlanta Dental College which position he filled with most marked ability.

As a Mason he was bright, was master of the lodge when he lived in La Grange and was a member of the lodge at College Park, his late place of residence, near Atlanta. He was a member of the Methodist Episcopal Church—South, Park street, West End. Dr. Chapple married Mrs. Annie Wagner Ware, of La Grange, Ga., in September, 1876, who survives him, together with a step-son, Mr. W. R. Ware of Atlanta, Ga., a daughter Mrs. Hilton H. Chandler of Waynesboro, Ga., and a son Mr. Robert Olin Chapple of Jacksonville, Fla.

Dr. Chapple was a man of most attractive personality, genial, generous, hightoned and honorable. As a reconteur he had few equals and was the life of the social gatherings of his confreres. As a writer on dental subjects, and his essays were numerous and varied, he was easily the most able and talented in the South. In his demise the profession at large has lost a most eminent and worthy member and his host of friends mourn his loss.

B. H. Teague.

---

Augusta, Ga., May 17th, 1906.

To the Family of Dr. Jas. A. Chapple, Atlanta, Ga.

We have just learned that Dr. Chapple has gone.

How sad these words are to us. No student has profited by his able teaching, who has not gone home with the most profound admiration and love for Dr. Chapple. None knew him but to love him. In his death we know that the whole dental fraternity has suffered a blow from which it will never recover, but we will try and condole ourselves when we realize that our profession is nobler, our aspirations higher, our learning is nearer perfection, and our determination to live upright lives more impressed upon us, by his life having touched ours.

To his family and friends we extend our most sincere sympathy.

Signed:

Walter C. Miller, Class 1900,
George M. Woodbury, Class 1900,
James A. Dobey, Class 1905,
L. G. Youmans, Class 1905,
J. T. Whitlaw, Class 1905,
R. H. Calhoun, Class 1904.

